# Eccrine Poroma: Pathogenesis, New Diagnostic Tools and Association with Porocarcinoma—A Review

**DOI:** 10.3390/diagnostics13162689

**Published:** 2023-08-16

**Authors:** Eirini Kyrmanidou, Christina Fotiadou, Christina Kemanetzi, Myrto-Georgia Trakatelli, Anastasia Trigoni, Aikaterini Patsatsi, Zoe Apalla, Elizabeth Lazaridou

**Affiliations:** 2nd Department of Dermatology, School of Medicine, Aristotle University of Thessaloniki, 54124 Thessaloniki, Greece; christinafotiadou@yahoo.com (C.F.); christina.kemanetzi@gmail.com (C.K.); mtrakatelli@hotmail.com (M.-G.T.); nastrigoni@yahoo.gr (A.T.); katerinapatsatsi@gmail.com (A.P.); zoimd@yahoo.gr (Z.A.); bethlaz@auth.gr (E.L.)

**Keywords:** eccrine poroma, eccrine porocarcinoma, gene fusion, dermoscopy, reflectance confocal microscopy

## Abstract

Eccrine poroma (EP) is a relatively rare benign adnexal neoplasm that usually affects elderly patients. Its pathogenesis is still under investigation, but recent gene studies have revealed gene fusions as key incidences resulting in oncogenetic pathways. It often presents as a solitary, firm papule, mostly asymptomatic, located on the soles or palms. Due to its clinical and dermoscopic variability, it is characterized as the great imitator. We performed a literature review, aiming to summarize current data on the pathogenetic mechanisms, new dermoscopic features, and novel diagnostic tools that may aid in early diagnosis and proper management of this rare adnexal tumor. Furthermore, we reviewed the possible pathogenetic associations between EP and its malignant counterpart, namely eccrine porocarcinoma. This systematic approach may aid in understanding the pathogenetic mechanisms and how to use novel histopathologic markers and imaging methods to overcome the diagnostic dilemma of this rare tumor.

## 1. Introduction

Cutaneous adnexal tumors include benign as well as malignant neoplasms. Their classification depends on the differentiation of the neoplastic cells towards typical adnexal structures of the skin: eccrine, apocrine, follicular, and sebaceous. They are relatively rare tumors, and clinicians often find it difficult to diagnose them. In this review, we will focus on eccrine poromas, the pathogenetic insights and new diagnostic tools that can be used to identify and diagnose this benign tumor.

Epidemiologically, eccrine and apocrine sweat gland neoplasms cover only 1% of all primary cutaneous lesions. Poromas are identified in approximately 10% of these lesions [1]. They usually occur in middle-aged to elderly patients with no clear sex predilection. The most common locations of the tumor are the palms and soles, the head and neck, as well as the lower extremities. However, less common locations have been described, such as the eyelids and the vulva [2,3]. Poroma is often referred to as the great imitator since, clinically and dermoscopically, it frequently shares features of common benign and/or malignant skin tumors, and thus histopathologic examination is needed to confirm the diagnosis. Depending on the localization of poroid cells, four histopathologic variants have been described: hidracanthoma simplex, eccrine poroma (EP), dermal duct tumor, and poroid hidradenoma. In this review, we will focus on EP, its morphology, pathophysiology, recent diagnostic advances, and the correlation with its malignant counterpart porocarcinoma.

## 2. Methods

We conducted a web-based literature research using the following terms: eccrine poroma; poroma; porocarcinoma; gene fusion; YAP/TAZ; YAP/TAZ expression; NUT; NUT fusion; Merkel cell polyomavirus; MCPyV; poroid cells; cuticular cells; dermoscopy; dermatoscopy; reflectance confocal microscopy; RCM; Line-field optical coherence tomography; LC-OCT; magnetic resonance imaging; MRI. We identified 147 relevant articles, and two of the researchers (E.K., C.F.) reviewed their content in consensus to finally decide which papers to include.

## 3. Pathogenesis—Molecular Features

Recent gene studies have revealed gene fusions as key incidences resulting in oncogenesis in several benign, intermediate, and aggressive tumors emerging from different histogenetic origins. Both poromas and porocarcinomas harbor activating mutations in HRAS, or fusions of YAP/TAZ, *YAP1-MAML2*, *YAP1-NUTM1*, or *WWTR1_NUTM1*. Gene fusions are defined as genes resulting from translocation, inversion, or deletion of two independent genes. Fusion genes can initiate the expression of proteins resulting in oncogenesis, since these fusions may frequently be insufficient in regulatory domains, inducing overexpression. YAP1 and WWTR1 (TAZ) genes are located on 11q22 and 3q25.1, respectively, and are responsible for encoding paralogous transcriptional regulators of TEAD-family DNA binding transcription factors [4]. Gene expression is controlled by the Hippo signaling pathway, a tumor suppressor pathway [5]. It is regulated by a kinase cascade, including, among others, the paralogous transcriptional coactivator proteins YAP and TAZ, hindering their entry into the nucleus [6]. YAP/TAZ expression is essential for cell proliferation and their overexpression results in organ overgrowth and leads almost inevitably to cancer [7]. Thus, during steady state multiple negative regulators consistently control YAP/TAZ, while when in regenerative or malignant growth YAP/TAZ is activated. Accordingly, YAP is highly expressed upon skin wounding, and on psoriatic skin it induces hyperproliferation and exuberant differentiation of epidermal keratinocytes [8,9]. In tumorigenesis, YAP/TAZ incites several oncogenes or inactivates tumor-suppressor genes supporting the survival of malignant cells. YAP1 or TAZ rearrangements have been detected in epithelioid hemangioendothelioma, pseudomyogenic hemangioendothelioma, ependymoma, epithelioid hemangioma, and, lately, in poroma and porocarcinoma [4,10,11,12,13].

Recently, Sekine et al. reported YAP1 fusions in 93/104 poroma cases (85%) and in 7/11 (67.6%) of porocarcinoma cases, investigating the possible tumorigenic roles of gene fusions. Namely, *YAP1-MAML2*, *MAML2-YAP-1*, *YAP1-NUTM1*, and *WWTR1-NUTM1* fusions were identified in 71, 48, 21, and 1 poromas, respectively. Moreover, among the 11 examined porocarcinomas, *YAP1-MAML2* and *YAP1-NUTM1* fusions were identified in 1 and 6 lesions, respectively. Overall, YAP1 fusions were expressed in 92% of poromas and 7% of porocarcinomas, with 1 poroma associated with a WWTR1 fusion. This suggests that poromas and porocarcinomas are related histogenetically and that a significant proportion of porocarcinomas may arise from the malignant transformation of preexisting poromas [14]. None of the other skin tumors examined, including 24 squamous cell carcinomas, 32 basal cell carcinomas, 5 cutaneous adenocarcinomas, 9 Merkel cell carcinomas, and 27 seborrheic keratoses, showed any evidence of recurring YAP1 fusions, as was reported in previous studies [15]. This suggests that YAP1 fusions are highly recurrent and specific to poromas and porocarcinomas among skin neoplasms. Thus, detection of protein products derived from YAP1 fusions could provide immediate and precise diagnosis.

Moreover, researchers speculated that since both genes, namely YAP1 and MAML2 are located in chromosome 11p and both *YAP1-MAML2* and *MAML2-YAP1* fusions often occur simultaneously in the same tumor, fusions related to YAP1 and MAML2 reflect an intrachromosomal inversion [14]. Additionally, NUTM1 fusion was detected in cutaneous poroid neoplasms, resulting in the expression of nuclear protein in testis (NUT) intranuclearly. This specific protein is exclusively present in testis and ovary germ cells, and its appearance in cutaneous poroid neoplasms could implement a potential specific marker aiding the diagnosis. Recently, Agaimy et al. described two porocarcinomas harboring *YAP1-NUTM1* fusions located at the external auditory canal, pointing out that NUT positivity identified with the use of immunohistochemistry is not pathognomonic for NUT carcinoma anymore [16]. Macagno et al. examined NUT expression in a cohort of 835 skin neoplasms and concluded that NUT is restricted to poroid tumors. In this study, NUT expression achieved 100% specificity in poroid tumors, but its absence cannot exclude the diagnosis (sensitivity 32%) [17].

The pathogenetic mechanism of skin tumors, especially basal cell carcinoma and squamous cell carcinoma, involves the effect of ultraviolet radiation. Ultraviolet UV-induced mutations can be confirmed in more than 70% of non-inherited BCCs gene mutations [18]. On the other hand, this seems not to be the key pathogenetic point in eccrine poroma, evolving primarily in the deep dermis which is less affected by ultraviolet radiation. Although NUT carcinomas are rare variants of squamous carcinoma, which harbor fusions on the NUTM1 gene on 15q14 chromosome, they more often reside in the upper respiratory and gastrointestinal tract and mediastinum. The simplicity of the cytogenetic profile of NUT carcinomas, resembling translocation-associated hematopoietic tumors, differs significantly from the mutagen-associated epithelial tumors that need years, numerous mutations, and genetic aberrations to provoke tumorigenesis. Given the fact that NUT carcinomas are highly aggressive, with a significant metastatic capacity, it may be speculated that either NUT carcinomas emerge from a single stem-like cell resulting from a single chromosomal event with migratory properties, or the NUT-fusion protein provokes epigenetic changes resulting in these cell characteristics [19]. Considering that squamous cell carcinomas on sun damaged skin need years to progress, it is doubtful that UV radiation can provoke a NUT positive eccrine poroma or porocarcinoma.

Recently, Meriläinen et al. tried to shed light on the pathogenetic role of Merkel cell polyomavirus (MCPyV) in both poroma and porocarcinoma [20]. It may be hypothesized that immunosuppression and opportunistic infections show a tumorigenic effect since multiple eccrine poromas and eccrine porocarcinomas evolve often in patients with malignancies and organ transplant recipients. In the pathogenesis of Merkel cell carcinoma, the pathogenetic pathway includes MCPyV infection and chronic ultraviolet exposure [21]. However, traces of MCPyV genomic material are identified in cancerous as well as in noncancerous tissues, and thus, a causative role of this virus can be speculated [22]. In the study, 17 porocarcinomas and 50 poromas were included and analyzed, and MCPyV was detected in 10% of eccrine poroma samples and in 18% of eccrine porocarcinoma samples, whilst in Merkel cell carcinoma samples, which served as the reference material, MCPyV was present in 64.5%. Namely, in Merkel cell carcinoma the virus DNA copy was at least 25 times higher, suggesting that MCPyV acts as a passenger virus in EP and EPC. Researchers conclude that MCPyV may not serve as an oncogenic driver for eccrine poroma or eccrine porocarcinoma.

## 4. Histopathology

Eccrine poromas are eccrine neoplasms that are histopathologically subdivided into: poroma, hidroacanthoma simplex, dermal duct tumor, and poroid hidradenoma. This classification partly depends on the location of the poroid cells in relation to the epidermis. Hidracanthoma simplex resides entirely intraepidermally, whereas dermal duct tumor is located entirely intradermally. A classic poroma consists of cords and clusters of neoplastic cells in connection with the epidermis and infiltration of the superficial dermis. Lastly, poroid hidradenoma, which is characterized as a single nodule or small number of nodules, resides in the dermis and has both solid and cystic segments.

All types of poromas share common histopathologic features:

(1) Poroid cells: their morphology resembles the cells of the peripheral layer of the most distal part of the eccrine and apocrine ducts. They have round nucleus and basophilic cytoplasm.
(2) Cuticular cells: in contrast to poroid cells, they appear larger with eosinophilic cytoplasm similar to luminar cells of the ductal part of eccrine and apocrine glands.

Eccrine poroma resides entirely in the acanthotic epidermis and arises from the peripheral cells of the intraepidermal part of the eccrine duct. From the lower epidermis it proliferates downwards into the dermis, forming broad anastomosing bands of epithelial cells. In comparison with epidermal keratinocytes, the tumor consists of uniform, cuboidal and smaller cells in size. Intercellular bridges connect the cells [23].

## 5. Clinical Features

Eccrine poroma presents as a solitary, firm papule, plaque or nodule sometimes surrounded by a rim. The surface may vary from smooth, shiny, and scaly to verrucous and papillomatous. Secondary ulcerations and erosions are not uncommon due to its location and shape. Most frequently EP is skin-colored, pink-red, and, when it is pigmented, it can be described as blue or even black [24]. Although poroma affects predominately volar skin, there have been cases reported on other areas, like the trunk, face, vulva, eyelids, and even subungually [3,25,26,27]. This predilection of acral surfaces could be justified by the fact that on these sites eccrine glands are denser and more numerous. However, EPs may appear even in areas with low density of eccrine glands, as mentioned above.

It is mostly asymptomatic and slow growing, but some patients report itch or, rarely, pain [28]. Although in most cases EPs appear as solitary tumors, cases of multiple lesions appearing as clusters have been reported. This condition is defined as eccrine poromatosis, a condition common in patients who were treated with radiotherapy and/or chemotherapy [29]. The pathogenesis of eccrine poromatosis is still not fully understood, but it is postulated that multiple eccrine poromas may be related to the long-term effects of chemotherapy and radiotherapy, rather than being regarded as a paraneoplastic phenomenon. In favor of this hypothesis, some authors report that toxic chemotherapy metabolites that are being secreted and concentrated in eccrine glands can subsequently lead to remodeling and the regeneration of cells predisposing to tumorigenesis. Toxic erythemas of chemotherapy, including neutrophilic eccrine hidradenitis (NEH) and syringosquamous metaplasia, are thought to share the above-mentioned etiopathogenesis [30,31].

## 6. Dermoscopy

**Figure 1:**
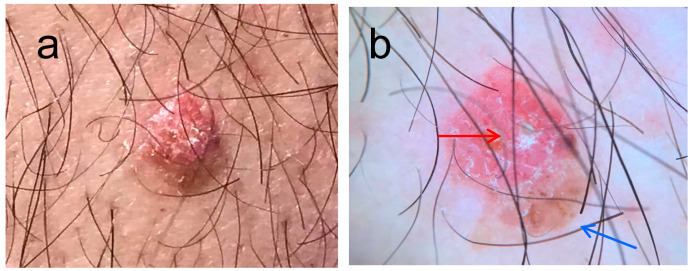
(**a**) Dome shaped lesion on the left arm of a 67 year old male, present for many years, clinical image, (**b**) on dermoscopy there is a vascular area (red arrow) with linear vessels and a brownish area resembling a blotch (blue arrow). The differential diagnosis in this case included irritated seborrhoic keratosis, amelanotic melanoma, and BCC.

Clinical diagnosis of EPs can be challenging. Especially in cases that appear on nonvolar areas, EPs can be misdiagnosed as malignant melanoma, basal cell carcinoma (BCC), or squamous cell carcinoma (SCC). Dermoscopy can aid towards the diagnosis of poroma, although specific dermoscopic features of EP have not yet been described. Dermoscopically poromas may share common features with pyogenic granulomas, seborrheic keratosis, nevi, malignant melanoma, BCC, and SCC (Figure 1).

In non-pigmented cases of poromas, the prominent dermoscopic feature that can shift towards diagnosis is the vascular pattern of the lesion. Most commonly appearing are polymorphic, glomerular, leaf- and flower-like, linear-irregular, and looped- or hairpin-like vascular structures. Amongst the abovementioned features, leaf- and flower-like structures appear to be characteristic for poromas. Moreover, when comparing to the arborizing vessels seen on basal cell carcinoma, vascular structures on poromas appear less in focus, indicating that the vessels in poromas are located much deeper in the dermis, providing hints for differential diagnosis.

Due to their heterogenic clinical appearance, the International Dermoscopy Society (IDS) conducted a multicenter analysis of high-quality and dermoscopic images of histopathologically verified poromas and corresponding controls. Pictures selected as controls had to be similar in clinical appearance, morphology, and anatomical location in the provided cases. Two hundred nineteen lesions, including 113 poromas, were selected for the study and evaluated for dermoscopic descriptions that unanimously, by all three blinded reviewers, were categorized into specific terminology. Thus, the described dermoscopic features that statistically appeared to be poroma-specific were white interlacing areas around vessels, branched vessels with rounded endings, yellow unstructured areas, and milky-red globules. Statistically, the diagnostic accuracy showed modest sensitivity and specificity (62.8% and 82%, respectively). Hence, highlighting the difficulty in diagnosing this polymorphous tumor, researchers insist that in order not to miss malignancy, any lesion with polymorphous vessels, ulceration, and/or shiny white structures should be biopsied to exclude amelanotic melanoma, melanoma metastasis, and SCC [32].

Undoubtedly, dermoscopic characteristics can often appear differently depending on specific anatomical locations, such as volar skin. To investigate possible differences in the dermoscopic characteristics of poromas on volar or non-volar skin Ha et al. [33] selected 20 patients with volar poroma and as many with non-volar location. Dermoscopic pictures were examined and associated with patterns specific for poroma, melanoma, and nonmelanoma skin cancer. Less than half of non-volar cases (45%) showed the typical clinical features, with a wide range of morphological variants and initial clinical diagnoses in this group. In patients with non-volar location, almost one-third of cases were clinically diagnosed as skin cancer, whereas no patient with volar poroma received this diagnosis. Moreover, non-volar poromas showed more often melanoma-associated dermoscopic features (45%), such as regression structures and a blue-white veil overlying a raised area compared to the volar poroma group. Likewise, poromas on non-volar skin, when present with atypical clinical features, showed dermoscopic patterns associated with basal cell carcinoma (40%) [33].

Chessa et al. [34] investigated the dermoscopic-histopathological correlations of eccrine poromas. The researchers examined 26 histopathologically diagnosed eccrine poromas, 10 of which were nonpigmented and 16 pigmented. During the clinical examination, all lesions were dome shaped with well-defined borders. Histopathologically, three of the four variants of eccrine poromas were identified in the case series, whereas poroid hydradenoma was not detected. Interestingly, several dermoscopic features were recurrent in each histopathological variant. Namely, in the four cases of nonpigmented hidroacanthoma simplex, the dermoscopic patterns identified were glomerular, linear irregular, flower-like, corkscrew vessels, and milia-like cysts, while the histopathological findings were circumscribed poroid cells confined within the epidermis. Additionally, in the five pigmented hidroacanthoma simplex lesions network-like structures, comedo-like openings, and milia cysts were identified dermoscopically, and the histopathologic pattern included circumscribed poroid cells confined within the epidermis plus an amount of melanin pigment within the epithelial component. When examining the six nonpigmented eccrine poromas, the dermoscopic pattern included glomerular, milky red globules, flower-like vessels, and dotted vessels, and the histopathologic pattern included poroid and cuticular cells located in the dermis that are continuous with the epidermis. In the pigmented eccrine poroma variant, the dermoscopic pattern was hairpin, linear irregular vessels, serpentine vessels, and milia-like cysts, while the histopathological pattern included increased amount of melanin present among the poroid cells in the dermis and epidermis. Lastly, the dermoscopic pattern consisted of leaf-like and flower-like vessels, whereas the histopathologic pattern in the two cases of pigmented dermal duct tumor included poroid cells that were aggregated in small discrete intradermal nodules [34]. With regard to dermal duct tumor, Tavoletti et al. recently described the dermoscopic findings of a nonpigmented dermal duct tumor as a red-orange nodule with well-defined borders, yellowish structureless areas, eccentrically located blue-grey ovoid nests, and a vascular pattern consisting of linear irregular and branched vessels of different length and caliber [35].

## 7. Other Diagnostic Tools

Recently, reflectance confocal microscopy (RCM) has been introduced as an additional diagnostic tool to improve our diagnostic accuracy towards malignant neoplasms. RCM allows in vivo visualization of structures, parallel to the skin surface, at several depths from stratum corneum to the superficial dermis. Di Tulio et al. recently performed a case–control study of 44 lesions: 11 EPs and 33 controls [36]. The control group consisted of lesions that are commonly included in the differential diagnosis of EP, such as basal cell carcinoma, nevus, seborrheic keratosis, melanoma, squamous cell carcinoma, dermatofibroma, and Spitz nevus. Comparison between the histopathologic examination and the RCM features were performed in all cases of EP. Tumor cells using RCM appear as small and uniform in size and shape, with brighter and more defined borders compared to the adjacent keratinocytes. As observed in histology, the tumor has well defined borders and structures that form regular cords without peripheral palisading. RCM identifies some non-refractile dark roundish spaces within the cords, and this represents areas of ductal differentiation on histology. Moreover, tumor cords are enclosed by a highly vascularized and abundant stroma. In cases of pigmented Eps, highly refractile large round cells and/or dendritic cells within the tumor can be identified corresponding to melanocytes. Researchers conclude that features such as “cords without palisading”, “dark holes”, “prominent vascularisation”, and “abundant stroma” are characteristics from the RCM examination that are linked to the diagnosis of EP. Lastly, absence of RCM features, typical of lesions commonly interfering in the diagnosis of EP, aided towards a correct diagnosis. Indeed, well defined tumor islands without peripheral palisading exclude basal cell carcinoma, whereas lack of features indicating melanocytic proliferation allows the exclusion of nevus or melanoma. RCM may be insufficient as a diagnostic tool when examining EPs on volar skin. As RCM has a penetration depth of up to 200μm, it is incapable of visualizing structures on acral skin, where the thickness of stratum corneum is greater. Likewise, hyperkeratosis and/or ulceration are also features that interfere with the diagnostic accuracy of RCM.

Line-field optical coherence tomography (LC-OCT) is another optical imaging method that combines vertical high-resolution visualization, similar to histopathological sections like RCM, but furthermore, it offers 3D reconstructions and of course overcomes the low penetration capacity of RCM by reaching up to 500 μm in depth. Maione et al., by using LC-OCT, correlated a case of EP with certain typical histological aspects [37]. The use of LC-OCT helped researchers rule out common skin tumors that are involved in the differential diagnosis of EP, such as melanoma and basal cell carcinoma. Melanocytic proliferation could be excluded by the absence of irregular honeycombed pattern, pagetoid spread, and dermal nests at vertical view [38]. Basal cell carcinoma was also excluded as branched lobules with the peculiar millefeuille pattern surrounded by a dark rim, features commonly present in this tumor, were absent [39].

Kawaguchi et al. conducted a study using magnetic resonance (MR) images and described features that can aid in diagnosing poromas from other skin tumors. The aim of the study was primarily to determine MR characteristics of poroma and its malignant counterpart porocarcinoma. Thus, pedunculated solid lesions that measure ≥ 30 mm in diameter and protrude into subcutaneous fat are two MR features that may be seen more often in porocarcinomas than in poromas. Additionally, intratumoral T1 hyperintensity is present in both poromas and porocarcinomas, and this can be related to the richly vascular stroma with dilated vessels within both tumors. Several MR characteristics have been described for skin tumors commonly included in the differential diagnosis of poromas. Namely, cutaneous squamous cell carcinoma is often presented with superficial ulceration, protrusion into subcutaneous fat and ill-defined borders. Cutaneous basal cell carcinoma is usually smaller in size and is not pedunculated, but T2 hyperintense foci may be present in both tumors [40]. Although in cutaneous malignant melanoma intratumoral T1 hypersensitivity is frequently present, indicating melanin or hemorrhage, intratumoral T2 hyperintense foci and pedunculation are absent [41]. Until today, MR imaging is not used as a diagnostic tool, but it can provide useful information preoperatively by determining skin tumor thickness and surgical margins.

## 8. Eccrine Porocarcinoma and Association

Eccrine porocarcinoma (ePC) is the malignant counterpart of eccrine poroma. Porocarcinoma is a rare malignant dermal duct tumor with an incidence of 0.004% which appears more often on the head and extremities. Due to the presence of squamous differentiation in ePC cells, the histopathological diagnosis imposes a challenge and, thus, it is often misdiagnosed as basal or squamous cell carcinoma. Hence, the low incidence rate of ePC may be an underestimation.

Pathogenetically, the appearance of the tumor is not fully understood, but a case series in 2021 reported that porocarcinomas may develop de novo or within a pre-existing poroma in 18% to 50% of cases [42]. Indeed, in some cases, it seems as if the presence of YAP1 fusions in both tumors could be the key event in oncogenesis. Parra et al. report a case of de novo PC with nuclear YAP1 (N-terminus) and NUTM1 immunohistochemical expression supporting common pathogenetic mechanisms in both poroma and PC [43]. One year later, Prieto-Granada et al. conducted a fusion gene analysis of three poromas and five porocarcinomas confirming the presence of YAP1 fusions, supporting furthermore the clinical observation of malignant transformation within poromas [44].

Clinical presentation of porocarcinoma includes an endoexophytic often ulcerated nodule, and, in some cases, it arises on a preexisting poroma. It is asymptomatic with erythematous to violaceous coloration and usually <2 cm. Differential diagnosis includes squamous cell carcinoma, malignant melanoma, basal cell carcinoma, seborrheic keratosis, cutaneous metastasis, traumatized nevus, chronic eczema, pyogenic granuloma, verruca vulgaris, angioma, and lipoma.

Signs causing us to suspect the malignant transformation of a preexisting poroma include spontaneous bleeding, itch, pain, ulceration, or sudden growth in a short period of time [45]. The variable clinical presentations and the wide differential diagnosis poses histological examination in the first line for diagnosing ePC. EPC characteristically consists of poromatous basaloid epithelial cells with ductal differentiation and cytologic pleomorphism. Additionally, tumor cells show nuclear hyperchromasia and increased mitotic activity. Ulceration and necrosis are often present. Although both tumors can share similar features, histopathologically eccrine poroma can be distinguished from eccrine porocarcinoma by lack of mitosis, infiltrative growth pattern, atypical cellular morphology, intratumoral necrosis, clear cell differentiation, or vascular invasion.

Porocarcinomas metastasize through the lymphatic system in ~25% of patients, with a mortality rate ranging from 65% to 80%, in such cases. Metastases are most commonly found in the adjacent lymph nodes (57.7%), followed by respiratory tract/lungs (12.8%), brain (9%), liver (9%), skin (5.8%), bones (3.2%), stomach (0.6%), and breast (0.6%) [46].

In a recent meta-analysis, Le et al. included 116 eccrine porocarcinomas from which in 25.0% the tumor cells showed squamous differentiation and 23.4% of the cases showed clear cell differentiation, which, however, did not serve as a prognostic factor. On the other hand, lymphovascular invasion and high mitotic activity were confirmed as negative prognostic factors. Additionally, lymphovascular invasion significantly increased the occurrence of local recurrence, regional recurrence, and distal metastasis [45]. Interestingly, a retrospective histopathological analysis of 19 PC cases confirmed the difficulty in diagnosing this tumor by the fact that seven (37%) were incorrectly diagnosed, i.e., five (26%) diagnosed as cutaneous SCC [47]. Immunohistochemical analysis can aid towards diagnosis as staining with carcinoembryonic antigen (CEA), epithelial membrane antigen (EMA), and additional histochemical staining with periodic acid-Schiff help visualize ductal structures, and thereby differentiate PCs from other types of tumors. Furthermore, PCs show some CD117 expressions and, although not specific to PC, in challenging cases, CD117 can aid in differentiating PC from cutaneous SCC [48].

## 9. Treatment

EP is a tumor with benign behavior, and its excision is curative. The superficial lesions can be treated via simple surgical excision, shave, or electrosurgically. Lesions expanding into deeper layers can be removed via simple surgical excision. On the contrary, PC as a malignant variant of poroma shows great recurrence rates after excision. Its removal can be performed via simple surgical excision, wide excision, Mohs micrographic surgery, radiation, and eventually amputation [49]. Surgical margins that decrease the recurrence rate are thought to be 3 to 10 mm. However, there is no evidence-based consensus on recommended excision margins. The local recurrence of porocarcinoma is estimated to occur in 20% to 35% of cases, lymph node metastasis in 20%, and solid organ metastasis in 10%. The high rate of metastasis to adjacent lymph nodes may support regional lymph node dissection, but there are still not enough data to recommend this procedure as routine. There are histopathologic features that are associated with poorer prognosis, such as >14 mitoses per high-power field, lymphovascular penetration, and tumor invasion > 7 mm [48]. In cases of EPC where surgery is not possible, primary radiotherapy can serve as a treatment option, but with variable results as no clinical studies confirm its efficacy. Additionally, chemotherapy using 5-fluorouracil or docetaxel may be used in cases of metastatic disease or recurrence [50].

## 10. Conclusions

EP is a benign tumor originating from the terminal portion of the sweat gland. It is characterized by versatility in its clinical and dermoscopic morphology, often mimicking features of other cutaneous neoplasms, and thus its diagnosis is challenging. Therefore, it is crucial to evaluate thoroughly the patient who presents a suspicious lesion in order to properly diagnose this tumor. Treatment usually consists of simple excision, but if the lesion recurs or presents with ulceration, bleeding, pain, or accelerated growth, eccrine porocarcinoma should be included in the differential diagnosis. Histopathologic examination and immunohistochemical staining can aid in identifying poromas and EPCs. Taking into consideration the rarity of this tumor, the clinical and dermoscopic difficulty in diagnosing poromas and tendency of poromas towards malignant transformation, it is advisable to examine histopathologically every suspicious nodular lesion especially in elderly patients.

## References

[1] Sawaya J.L., Khachemoune A. (2014). Poroma: A review of eccrine, apocrine, and malignant forms. Int. J. Dermatol..

[2] McCoskey M., Neerukonda V.K., Hatton M.P., Wolkow N. (2021). Eccrine poroma of the eyelid. Orbit.

[3] Karpathiou G., Mobarki M., Corsini T., Douchet C., Chauleur C., Peoc’h M. (2019). Eccrine Poroma of the Vulva. Am. J. Dermatopathol..

[4] Kao Y.-C., Lee J.-C., Zhang L., Sung Y.-S., Swanson D., Hsieh T.-H., Liu Y.-R., Agaram N.P., Huang H.-Y., Dickson B.C. (2020). Recurrent YAP1 and KMT2A Gene Rearrangements in a Subset of MUC4-negative Sclerosing Epithelioid Fibrosarcoma. Am. J. Surg. Pathol..

[5] Ma S., Meng Z., Chen R., Guan K.-L. (2019). The Hippo Pathway: Biology and Pathophysiology. Annu. Rev. Biochem..

[6] Rognoni E., Walko G. (2019). The Roles of YAP/TAZ and the Hippo Pathway in Healthy and Diseased Skin. Cells.

[7] Zanconato F., Cordenonsi M., Piccolo S. (2016). YAP/TAZ at the Roots of Cancer. Cancer Cell.

[8] Jia J., Li C., Yang J., Wang X., Li R., Luo S., Li Z., Liu J., Liu Z., Zheng Y. (2018). Yes-associated protein promotes the abnormal proliferation of psoriatic keratinocytes via an amphiregulin dependent pathway. Sci. Rep..

[9] Walko G., Woodhouse S., Oliveira Pisco A., Rognoni E., Liakath-Ali K., Lichtenberger B.M., Mishra A., Telerman S., Viswanathan P., Logtenberg M. (2019). 581 A genome-wide screen identifies YAP/WBP2/TEAD interplay conferring growth advantage on human epidermal stem cells. J. Investig. Dermatol..

[10] Patel N.R., Salim A.A., Sayeed H., Sarabia S.F., Hollingsworth F., Warren M., Jakacky J., Tanas M., Oliveira A.M., Rubin B.P. (2015). Molecular characterization of epithelioid haemangioendotheliomas identifies novel *WWTR1CAMTA1* fusion variants. Histopathology.

[11] Antonescu C.R., Chen H.-W., Zhang L., Sung Y.-S., Panicek D., Agaram N.P., Dickson B.C., Krausz T., Fletcher C.D. (2014). ZFP36-FOSB fusion defines a subset of epithelioid hemangioma with atypical features. Genes Chromosomes Cancer.

[12] Panagopoulos I., Lobmaier I., Gorunova L., Heim S. (2019). Fusion of the Genes WWTR1 and FOSB in Pseudomyogenic Hemangioendothelioma. Cancer Genom. Proteom..

[13] Pajtler K., Wei Y., Okonechniov K., Vouri M., Sahm F., Bunt J., Jones D., Korshunov A., Lichter P., Pfister S. (2019). Epen-06. yap1 subgroup supratentorial ependymoma requires tead and nuclear factor i-mediated transcriptional programs for tumorigenesis. Neuro-Oncology.

[14] Sekine S., Kiyono T., Ryo E., Ogawa R., Wakai S., Ichikawa H., Suzuki K., Arai S., Tsuta K., Ishida M. (2019). Recurrent YAP1-MAML2 and YAP1-NUTM1 fusions in poroma and porocarcinoma. J. Clin. Investig..

[15] Debaugnies M., Sánchez-Danés A., Rorive S., Raphaël M., Liagre M., Parent M.-A., Brisebarre A., Salmon I., Blanpain C. (2018). YAP and TAZ are essential for basal and squamous cell carcinoma initiation. EMBO Rep..

[16] Agaimy A., Tögel L., Haller F., Zenk J., Hornung J., Märkl B. (2020). YAP1-NUTM1 Gene Fusion in Porocarcinoma of the External Auditory Canal. Head Neck Pathol..

[17] Macagno N., Kervarrec T., Sohier P., Poirot B., Haffner A., Carlotti A., Balme B., Castillo C., Jullie M.-L., Osio A. (2021). NUT Is a Specific Immunohistochemical Marker for the Diagnosis of YAP1-NUTM1-rearranged Cutaneous Poroid Neoplasms. Am. J. Surg. Pathol..

[18] Hernandez L.E., Mohsin N., Levin N., Dreyfuss I., Frech F., Nouri K. (2022). Basal cell carcinoma: An updated review of pathogenesis and treatment options. Dermatol. Ther..

[19] French C.A. (2018). NUT Carcinoma: Clinicopathologic features, pathogenesis, and treatment. Pathol. Int..

[20] Meriläinen A.S., Sihto H., Koljonen V. (2021). Merkel cell polyomavirus is a passenger virus in both poroma and porocarcinoma. J. Cutan. Pathol..

[21] Yang J.F., You J. (2022). Merkel cell polyomavirus and associated Merkel cell carcinoma. Tumour Virus Res..

[22] Csoboz B., Rasheed K., Sveinbjørnsson B., Moens U. (2020). Merkel cell polyomavirus and non-Merkel cell carcinomas: Guilty or circumstantial evidence?. APMIS.

[23] Deckelbaum S., Touloei K., Shitabata P.K., Sire D.J., Horowitz D. (2013). Eccrine poromatosis: Case report and review of the literature. Int. J. Dermatol..

[24] Xu M., Chen Y., Xiong J., Xue R., Yin J., Yang W. (2021). Pigmented poroma showing unique pineapple-like dermoscopic appearance with target network-like structure. Int. J. Dermatol..

[25] Ahuja S., Kaur A., Goel M., Raghuvanshi S., Arya A. (2019). Eccrine intraepidermal poroma of the eyelid. Indian J. Ophthalmol..

[26] Lim G.H., Abd Rashid F., Wong A. (2019). Eccrine poroma of the nipple: The first reported case. BMJ Case Rep..

[27] Yorulmaz A., Aksoy G.G., Ozhamam E.U. (2020). A Growing Mass under the Nail: Subungual Eccrine Poroma. Ski. Appendage Disord..

[28] Shalom A., Schein O., Landi C., Marghoob A., Carlos B., Scope A. (2012). Dermoscopic Findings in Biopsy-Proven Poromas. Dermatol. Surg..

[29] Mayo T.T., Kole L., Elewski B. (2015). Eccrine Poromatosis: Case Report, Review of the Literature, and Treatment. Ski. Appendage Disord..

[30] Valdebran M.A., Hong C., Cha J. (2018). Multiple Eruptive Eccrine Poromas Associated with Chemotherapy and Autologous Bone Marrow Transplantation. Indian Dermatol. Online J..

[31] Choi E.C.E., Lim J.H.L. (2021). Eccrine poromatosis and polychemotherapy. Int. J. Dermatol..

[32] Marchetti M.A., Marino M.L., Virmani P., Dusza S.W., Marghoob A.A., Nazzaro G., Lallas A., Landi C., Cabo H., Quiñones R. (2018). Dermoscopic features and patterns of poromas: A multicentre observational case-control study conducted by the International Dermoscopy Society. J. Eur. Acad. Dermatol. Venereol. JEADV.

[33] Ha D.-L., Lee G.-W., Shin K., Kim H.-S., Ko H.-C., Kim B.-S., Kim M.-B. (2020). Characteristic Clinical and Dermoscopic Features of Nonvolar Poroma. J. Cutan. Med. Surg..

[34] Chessa M.A., Patrizi A., Baraldi C., Fanti P.A., Barisani A., Vaccari S. (2019). Dermoscopic-Histopathological Correlation of Eccrine Poroma: An Observational Study. Dermatol. Pract. Concept..

[35] Tavoletti G., Avallone G., Maronese C., Boggio F., Marzano A., Nazzaro G. (2023). Dermoscopy of dermal duct tumour. Australas. J. Dermatol..

[36] Di Tullio F., Mandel V.D., Ignazio S., Cinotti E., Kaleci S., Ciardo S., Peccerillo F., Longo C., Farnetani F., Pellacani G. (2022). The role of reflectance confocal microscopy in the diagnosis of eccrine poroma: A retrospective case–control study. Exp. Dermatol..

[37] Maione V., Bighetti S., Bettolini L., Zambelli C., Calzavara-Pinton P. (2023). The role of line-field confocal optical coherence tomography (LC-OCT) in the diagnosis of eccrine poroma: A case report. Australas. J. Dermatol..

[38] Schuh S., Ruini C., Perwein M.K.E., Daxenberger F., Gust C., Sattler E.C., Welzel J. (2022). Line-Field Confocal Optical Coherence Tomography: A New Tool for the Differentiation between Nevi and Melanomas?. Cancers.

[39] Suppa M., Fontaine M., Dejonckheere G., Cinotti E., Yélamos O., Diet G., Tognetti L., Miyamoto M., Orte Cano C., Perez-Anker J. (2020). Line-field confocal optical coherence tomography of basal cell carcinoma: A descriptive study. J. Eur. Acad. Dermatol. Venereol..

[40] Kawaguchi M., Kato H., Tomita H., Hara A., Suzui N., Miyazaki T., Matsuyama K., Seishima M., Matsuo M. (2020). Magnetic Resonance Imaging Findings Differentiating Cutaneous Basal Cell Carcinoma from Squamous Cell Carcinoma in the Head and Neck Region. Korean J. Radiol..

[41] Kawaguchi M., Kato H., Tomita H., Hara A., Suzui N., Miyazaki T., Matsuyama K., Seishima M., Matsuo M. (2020). MR imaging findings for differentiating cutaneous malignant melanoma from squamous cell carcinoma. Eur. J. Radiol..

[42] Olmos Nieva C.C., Samaniego González E., González Morán M.A., Rodríguez Prieto M.A. (2021). Eccrine Porocarcinoma: A Clinical and Histologic Description of a Series of 11 Cases Treated at the University Hospital Complex in Leon, Spain. Actas Dermo-Sifiliográficas.

[43] Parra O., Kerr D.A., Bridge J.A., Loehrer A.P., Linos K. (2020). A case of *YAP1* and *NUTM1* rearranged porocarcinoma with corresponding immunohistochemical expression: Review of recent advances in poroma and porocarcinoma pathogenesis with potential diagnostic utility. J. Cutan. Pathol..

[44] Prieto-Granada C., Morlote D., Pavlidakey P., Rodriguez-Waitkus P., Ramirez C., Florento E., Swensen J., Gatalica Z., Stevens T.M. (2021). Poroid adnexal skin tumors with *YAP1* fusions exhibit similar histopathologic features: A series of six *YAP1* rearranged adnexal skin tumors. J. Cutan. Pathol..

[45] Le N.S., Janik S., Liu D.T., Grasl S., Faisal M., Pammer J., Schickinger-Fischer B., Hamzavi J.S., Seemann R., Erovic B.M. (2020). Eccrine porocarcinoma of the head and neck: Meta-analysis of 120 cases. Head Neck.

[46] Salih A.M., Kakamad F.H., Baba H.O., Salih R.Q., Hawbash M.R., Mohammed S.H., Othman S., Saeed Y.A., Habibullah I.J., Muhialdeen A.S. (2017). Porocarcinoma; presentation and management, a meta-analysis of 453 cases. Ann. Med. Surg..

[47] Belin E., Ezzedine K., Stanislas S., Lalanne N., Beylot-Barry M., Taieb A., Vergier B., Jouary T. (2011). Factors in the surgical management of primary eccrine porocarcinoma: Prognostic histological factors can guide the surgical procedure. Br. J. Dermatol..

[48] Joshy J., Mistry K., Levell N.J., Bodegraven B., Vernon S., Rajan N., Craig P., Venables Z.C. (2022). Porocarcinoma: A review. Clin. Exp. Dermatol..

[49] Brown C.W., Dy L.C. (2008). Eccrine porocarcinoma. Dermatol. Ther..

[50] Le H.M.L., Faugeras L., De Moor V., Fervaille C., Vander Borght T., Collette F., D’Hondt L. (2021). Eccrine Porocarcinoma: A Challenging Diagnostic and Therapeutic Tumoral Entity. Case Rep. Oncol..

